# An interpretable machine learning model for early prediction of Escherichia coli infection in ICU patients

**DOI:** 10.3389/fcimb.2025.1682764

**Published:** 2025-11-24

**Authors:** Shu Yang, Laiyu Zou, Huixin Liang, Xiaohong Xu, Xiaoling Chen

**Affiliations:** 1 Institute of Hematology, Fujian Medical University Union Hospital, Fuzhou, China; 2 Department of Infectious Disease, Fujian Medical University Union Hospital, Fuzhou, China; 3 Department of Laboratory Medicine, Fujian Medical University Union Hospital, Fuzhou, China

**Keywords:** Escherichia coli infection, machine learning, support vector machine, predictive model, intensive care unit

## Abstract

**Background:**

Early and accurate identification of *Escherichia coli* (*E. coli*) infection in intensive care unit (ICU) patients remains challenging butmay improve clinical outcomes if addressed effectively. This study aimed to develop and validate an interpretable machine learning model for early prediction of *E. coli* infection at ICU admission.

**Methods:**

This retrospective study was conducted using the Medical Information Mart for Intensive Care IV (MIMIC-IV) database. Adult patients (aged 18–100 years) with their first ICU admission and a length of stay ≥24 hours were included. *E. coli* infection was identified based on microbiological results and diagnostic codes. Missing data were imputed using the missForest algorithm. Feature selection was performed with Boruta and least absolute shrinkage and selection operator (LASSO), and intersecting variables were used for model construction. Eight machine learning models, logistic regression, k-nearest neighbors, decision tree, random forest, extreme gradient boosting, light gradient boosting machine, support vector machine (SVM), and neural network, were developed. Model performance in the validation cohort was assessed using area under the receiver operating characteristic curve (AUC) with 95% confidence interval (CI), sensitivity, specificity, F1 score, calibration curves, decision curve analysis (DCA), and clinical impact curves (CIC). Model interpretability was evaluated with Shapley additive explanations (SHAP).

**Results:**

A total of 52, 554 ICU patients were analyzed, of whom 4, 157 (7.9%) had *E. coli* infection. Twenty-eight intersecting variables were selected for modeling. Among all models, the SVM achieved the highest discrimination (AUC = 0.745, 95% CI: 0.726-0.764), followed by random forest (AUC = 0.742) and extreme gradient boosting (AUC = 0.739). Calibration and decision analyses indicated robust model calibration and clinical utility. SHAP analysis identified gender, age, sepsis, sedative use, and potassium level as the most influential predictors. A web-based tool was developed to enable real-time clinical risk estimation and individualized interpretability.

**Conclusions:**

An interpretable SVM-based machine learning model was developed and validated for early prediction of *E. coli* infection in ICU patients, demonstrating good discrimination, calibration, and potential clinical benefit. The associated online tool provides transparent, individualized risk predictions and may facilitate timely clinical decision-making.

## 1 Introduction

Nosocomial infections represent a major clinical and public health challenge in intensive care units (ICUs) worldwide. Critically ill patients are especially vulnerable to hospital-acquired infections due to underlying comorbidities, invasive procedures, and prolonged exposure to antibiotics and hospital environments (Etemad et al., 2021; Kollef et al., 2021). Recent epidemiological studies reported that ICU-acquired infections were associated with significant morbidity and mortality, with overall in-hospital mortality rates for ICU-acquired infections approaching or exceeding 40% in some cohorts (Etemad et al., 2021). Among the pathogens responsible for ICU infections, *Escherichia coli* (*E. coli*) has consistently emerged as one of the most common and clinically significant organisms. Recent large-scale studies demonstrate that *E. coli* is a leading cause of ICU-associated urinary tract infections, intra-abdominal infections, and bloodstream infections, accounting for a substantial proportion of all Gram-negative isolates in both medical and surgical ICU settings (Yali et al., 2014; Koksal et al., 2017; Sommerstein et al., 2021; Le Goff et al., 2025). The increasing prevalence of antimicrobial-resistant *E. coli*, including ESBL- and carbapenemase-producing strains, has further complicated infection management and empirical therapy in ICU settings (Assawatheptawee et al., 2021; Zhang et al., 2023). The increasing frequency of *E. coli*-related sepsis, high mortality in vulnerable populations, and the growing threat of antibiotic resistance highlight the importance of accurate and early risk stratification for *E. coli* infection in critically ill patients.

Accurate risk assessment and early identification of ICU patients at high risk for *E. coli* infection are crucial for timely intervention and improved clinical outcomes. Recent studies have explored risk factors, prediction models, and laboratory indicators for *E. coli* infection and its outcomes in various clinical populations. For example, a large electronic health record-based machine learning study found that older age, frequent urinary tract infections, and recent hospital visits were significant predictors of invasive *E. coli* disease, highlighting the potential of data-driven approaches for individualized risk assessment (Clarke et al., 2024). In another study from Chang et.al., prior exposure to carbapenems, chronic liver disease, and regular dialysis were identified as independent risk factors for carbapenem-nonsusceptible *E. coli* bacteremia (Chang et al., 2011). Clinical characteristics and comorbidities also played important roles. In older adults, bile duct stone, kidney stone, and urinary tract infection have been shown to significantly increase the risk of *E. coli* bloodstream infection, while prior use of cephalosporins and invasive procedures were linked to ESBL-producing *E. coli* infection (Chen et al., 2021). Moreover, analysis of adult sepsis patients with *E. coli* infection revealed that elevated red cell distribution width (RDW) and hematocrit (HCT) were associated with higher in-hospital mortality, and their predictive value surpassed that of conventional scores such as SOFA and APACHE II (Song et al., 2022). However, robust, clinically interpretable machine learning models specifically tailored to predicting *E. coli* infection risk in ICU populations remain limited.

Machine learning methods offer significant advantages over traditional statistical approaches in handling high-dimensional, heterogeneous clinical data, uncovering complex non-linear relationships, and enabling individualized risk prediction. These data-driven algorithms can automatically learn from large-scale electronic health records, integrate diverse variables, and improve predictive accuracy beyond conventional scoring systems (Kim et al., 2022; Li et al., 2022). Machine learning methods have shown remarkable promise in clinical prediction tasks. Machine learning models have been successfully developed for predicting infection (Tabah et al., 2023), sepsis (Wang et al., 2021), and organ dysfunction (Fan et al., 2023) in critical care settings, offering improved accuracy compared to traditional approaches. Furthermore, in the field of microbiology, machine learning has been successfully applied to predict antimicrobial resistance (AMR) from whole-genome sequencing data (Ren et al., 2022; Zhang et al., 2025).

In this study, we developed and validated an interpretable machine learning model for early prediction of E. coli infection in ICU patients using the large-scale MIMIC-IV database. A total of 52, 554 patients were analyzed, and 28 clinically relevant features were identified through Boruta and LASSO feature selection. Among all eight models, the SVM model demonstrated the best discrimination, with robust calibration and clinical utility supported by decision analyses. SHAP interpretability revealed key predictors such as gender, age, sepsis, sedative use, and potassium level. To facilitate clinical application, we further deployed the optimal model as an interactive web-based tool, enabling real-time individualized risk prediction and supporting early infection surveillance in ICU practice.

## 2 Data and methods

### 2.1 Data source

The data for this study were extracted from the Medical Information Mart for Intensive Care IV (MIMIC-IV) database (Johnson et al., 2023). MIMIC-IV is a large, publicly available, de-identified database comprising comprehensive clinical data of patients admitted to the intensive care units (ICUs) at the Beth Israel Deaconess Medical Center (Boston, MA, USA) from 2008 to 2022. The database includes demographic information, laboratory measurements, vital signs, medications, procedures, and detailed patient outcomes, providing a valuable resource for clinical research and model development. Access to the MIMIC-IV database was granted after completion of the required Collaborative Institutional Training Initiative (CITI) program (Record ID: 14220853). This study was conducted in accordance with the ethical standards laid down in the Declaration of Helsinki and was approved by the Institutional Review Boards (IRBs) of both the Massachusetts Institute of Technology (MIT) and Beth Israel Deaconess Medical Center. As the data in MIMIC-IV are fully de-identified, informed consent was waived for all participants.

### 2.2 Patient selection

**Figure 1:**
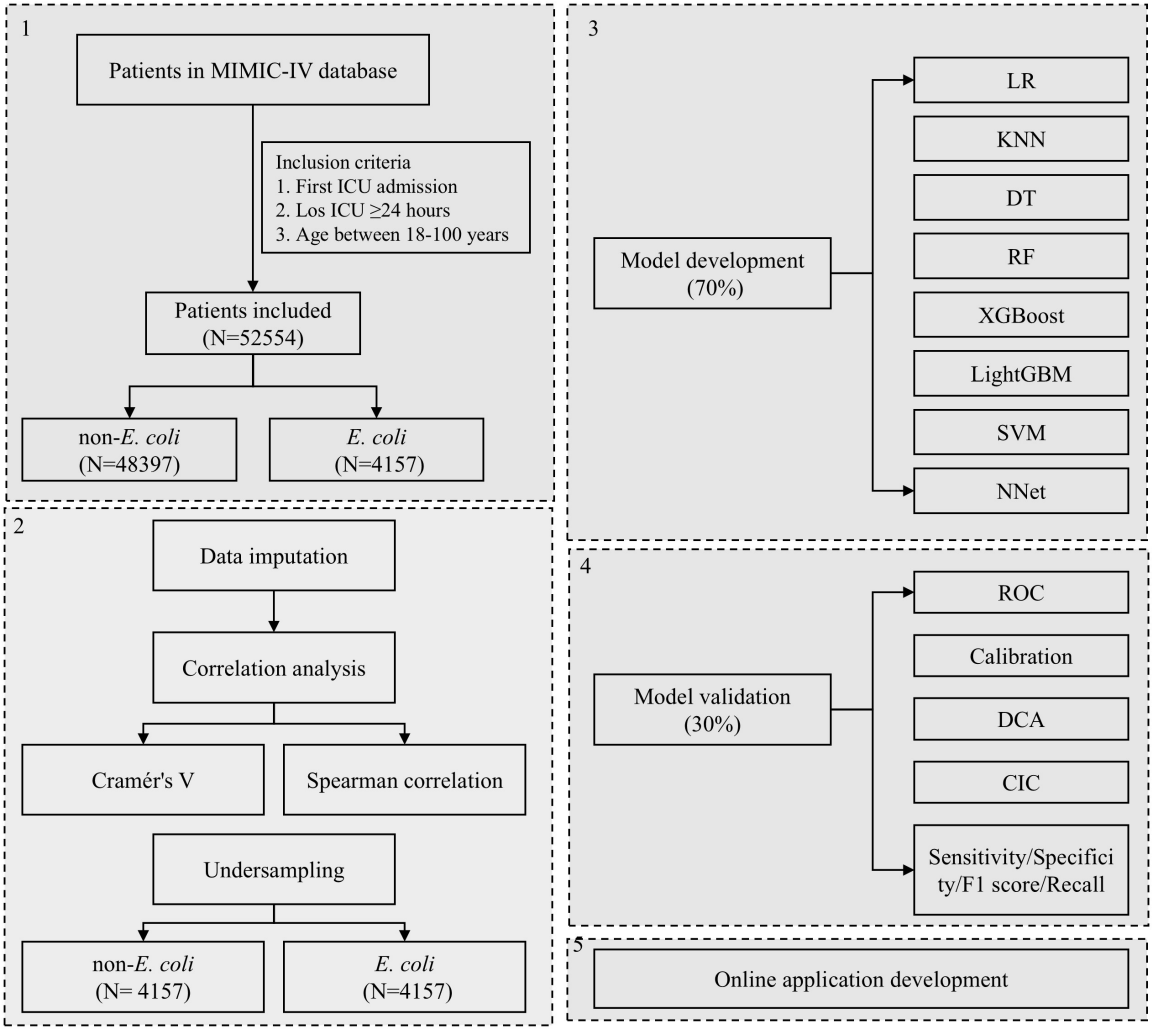
The flowchart of study design. ICU, Intensive Care Unit; LOS, Length of Stay; LR, Logistic Regression; KNN, K-Nearest Neighbors; DT, Decision Tree; RF, Random Forest; XGBoost, Extreme Gradient Boosting; LightGBM, Light Gradient Boosting Machine; SVM, Support Vector Machine; NNet, Neural Network; ROC, Receiver Operating Characteristic; DCA, Decision Curve Analysis; CIC, Clinical Impact Curve.

The overall flowchart of this study is presented in **Figure 1**. Patients were identified from the MIMIC-IV database using structured query language (SQL) based on the following inclusion criteria: (1) first ICU admission; (2) length of ICU stay of at least 24 hours; and (3) age between 18 and 100 years at the time of ICU admission. Patients with *Escherichia coli* infection were identified according to microbiological test results and International Classification of Diseases (ICD) codes documented during their hospitalization.

### 2.3 Variable extraction

Baseline data at ICU admission were extracted from the MIMIC-IV database using structured query language (SQL). The following variables were collected: (1) General and demographic information: age, gender, race, marital status, weight, height, input amount sum, output amount sum, liquid balance value, and urine output sum. (2) Vital signs: heart rate, systolic blood pressure (SBP), diastolic blood pressure (DBP), respiratory rate, and temperature. (3) Laboratory tests: white blood cell count (WBC), absolute neutrophil count, absolute monocyte count, absolute lymphocyte count, absolute eosinophil count, absolute basophil count, percentage of neutrophils, monocytes, lymphocytes, eosinophils, and basophils, C-reactive protein (CRP), red blood cell count (RBC), hemoglobin, hematocrit, red cell distribution width (RDW), platelet count, albumin, gamma-glutamyl transferase (GGT), alanine aminotransferase (ALT), alkaline phosphatase (ALP), aspartate aminotransferase (AST), total bilirubin, blood urea nitrogen (BUN), creatinine, lactate dehydrogenase (LDH), calcium, potassium, sodium, glucose, chloride, anion gap, D-dimer, fibrinogen, international normalized ratio (INR), prothrombin time (PT), partial thromboplastin time (PTT), partial pressure of oxygen (PO_2_), partial pressure of carbon dioxide (PCO_2_), PaO_2_/FiO_2_ ratio, oxygen saturation (SO_2_), lactate, pH, bicarbonate, and base excess. (4) Comorbidities: myocardial infarction, congestive heart failure, cerebrovascular disease, chronic pulmonary disease, liver disease, renal disease, diabetes, hypertension, malignant cancer, acquired immune deficiency syndrome (AIDS), acute kidney injury (AKI), AKI stage, sepsis, and delirium. (5) Clinical treatments: systemic glucocorticoids, inhaled corticosteroids (ICS), immunosuppressors, biological agents, vasopressors, proton pump inhibitors (PPIs), neuromuscular blocking agents (NMBA), sedatives, opioids, nonsteroidal anti-inflammatory drugs (NSAIDs), statins, invasive ventilation, noninvasive ventilation, continuous renal replacement therapy (CRRT), invasive lines, tubes, enteral nutrition, parenteral nutrition, antibiotic use, duration of invasive ventilation, duration of noninvasive ventilation, and days on CRRT. (6) Clinical scoring systems: Acute Physiology Score III (APS III), Logistic Organ Dysfunction Score (LODS), Oxford Acute Severity of Illness Score (OASIS), Systemic Inflammatory Response Syndrome (SIRS), Sequential Organ Failure Assessment (SOFA), Simplified Acute Physiology Score II (SAPS II), Glasgow Coma Scale (GCS), and Charlson comorbidity index. (7) Outcome information: length of hospital stay, hospital mortality, ICU stay and outcome at 14, 28, 30, 90, and 365 days.

### 2.4 Statistical analysis

Variables with more than 25% missing values in the total dataset were excluded from the analysis. The details of missing data were shown in **Supplementary Table 1**. And the total dataset was then randomly divided into a training set and a validation set at a 7:3 ratio. For variables with less than 25% missing data, the missForest algorithm, a non-parametric imputation method based on random forests, was used to impute missing values in both training and validation datasets.

In the training set, correlation analysis was performed. For continuous variables, Spearman’s rank correlation coefficients were calculated to assess the pairwise associations (**Supplementary Figure 1**). For any two continuous variables with a correlation coefficient greater than 0.6, the variable with the weaker association with the outcome (*Escherichia coli* infection) was excluded. For categorical variables, Cramér’s V was used to evaluate the strength of association between pairs of variables (**Supplementary Figure 2**). If two categorical variables had a Cramér’s V greater than 0.5, the variable less correlated with the outcome was removed.

Given the substantial class imbalance between the two outcome groups, an undersampling technique was employed to balance the training dataset. Undersampling involves randomly removing samples from the majority class to ensure that the number of cases in each class is comparable, thereby reducing potential bias and improving the robustness of model training.

Feature selection and model development were performed on the training set. Two methods, least absolute shrinkage and selection operator (LASSO) regression and the Boruta algorithm, were used for variable selection, and only variables identified by both methods were retained for model construction. Eight machine learning algorithms were developed using the selected features: logistic regression (LR), K-nearest neighbors (KNN), decision tree (DT), random forest (RF), extreme gradient boosting (XGBoost), light gradient boosting machine (LightGBM), support vector machine (SVM), and neural network (NNet). For each model, hyperparameter tuning was conducted using grid search in conjunction with k-fold cross-validation (typically 5- or 10-fold, depending on model type) to optimize predictive performance and prevent overfitting. The detailed hyperparameter settings and optimal configurations for all eight models were summarized in **Supplementary Table 2**. Model performance was evaluated on the validation set using several metrics: the area under the receiver operating characteristic curve (AUC) with confidence interval (CI), sensitivity, specificity, F1 score, balance accuracy, area under the precision-recall curve (AUPRC)calibration curves, decision curve analysis (DCA), and clinical impact curves (CIC). The interpretability of the optimal model was assessed using Shapley additive explanations (SHAP). A web-based clinical decision support application was developed using the Shiny framework to facilitate clinical application of the predictive model.

Descriptive statistics were used to summarize baseline characteristics of the study population. Continuous variables were reported as mean ± standard deviation (SD) for normally distributed data or median (interquartile range, IQR) for non-normally distributed data. Categorical variables were presented as frequencies and percentages. Group comparisons were performed using the Student’s t-test or the Mann-Whitney U test for continuous variables, as appropriate, and the chi-square test or Fisher’s exact test for categorical variables. All hypothesis tests were two-tailed, and a p-value < 0.05 was considered statistically significant. Statistical analyses and model development were performed using R software (version 4.4.2).

## 3 Results

### 3.1 Baseline characteristics of patients between non-*E. coli* group and *E. coli* group

A total of 52, 554 ICU patients were included, with 4, 157 (7.9%) diagnosed with *Escherichia coli* infection. Detailed baseline characteristics for the non-*E. coli* and *E. coli* groups were presented in **Supplementary Table 3**.

Compared to the non-*E. coli* group, patients in the *E. coli* group were older (mean age 70.1 vs. 65.1 years), more likely to be female, and had a higher proportion of widowed individuals. The *E. coli* group exhibited greater disease severity, with higher rates of congestive heart failure, cerebrovascular disease, liver and renal disease, diabetes, malignant cancer, delirium, and sepsis. Laboratory findings indicated that *E. coli* patients had higher levels of WBC, RDW, BUN, creatinine, anion gap, and INR, but lower hemoglobin, hematocrit, and calcium. They also had higher SOFA, SAPSII, APSIII, OASIS, and Charlson comorbidity index scores, indicating increased clinical complexity. Clinically, the *E. coli* group experienced longer hospital stays (median 16.2 vs. 10.5 days), higher rates of hospital mortality (13.0% vs. 10.4%), and increased use of vasopressors, PPIs, CRRT, enteral and parenteral nutrition, and antibiotics. Notably, short- and long-term ICU mortality (14, 30, 90, and 365-day) were consistently greater among *E. coli* patients.

### 3.2 Feature selection

**Figure 2:**
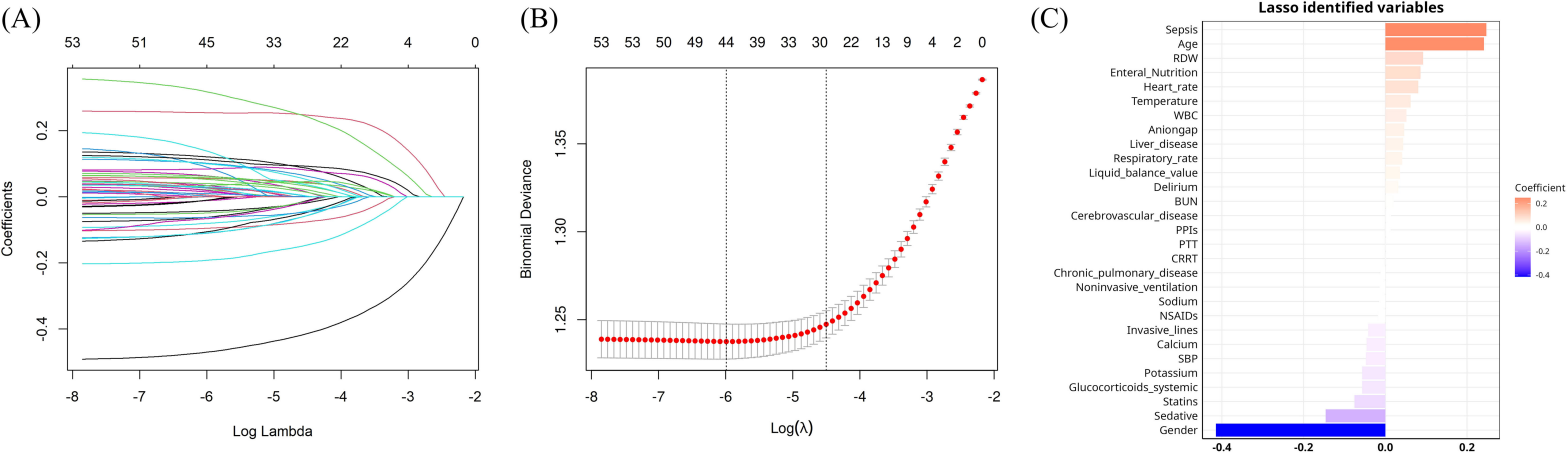
Lasso regression-based variable screening. **(A)** LASSO coefficient profiles of candidate variables. **(B)** Ten-fold cross-validation plot for selecting the optimal penalty parameter (λ) in the LASSO model. **(C)** Bar plot of variables selected by LASSO regression with their corresponding coefficients.

**Figure 3:**
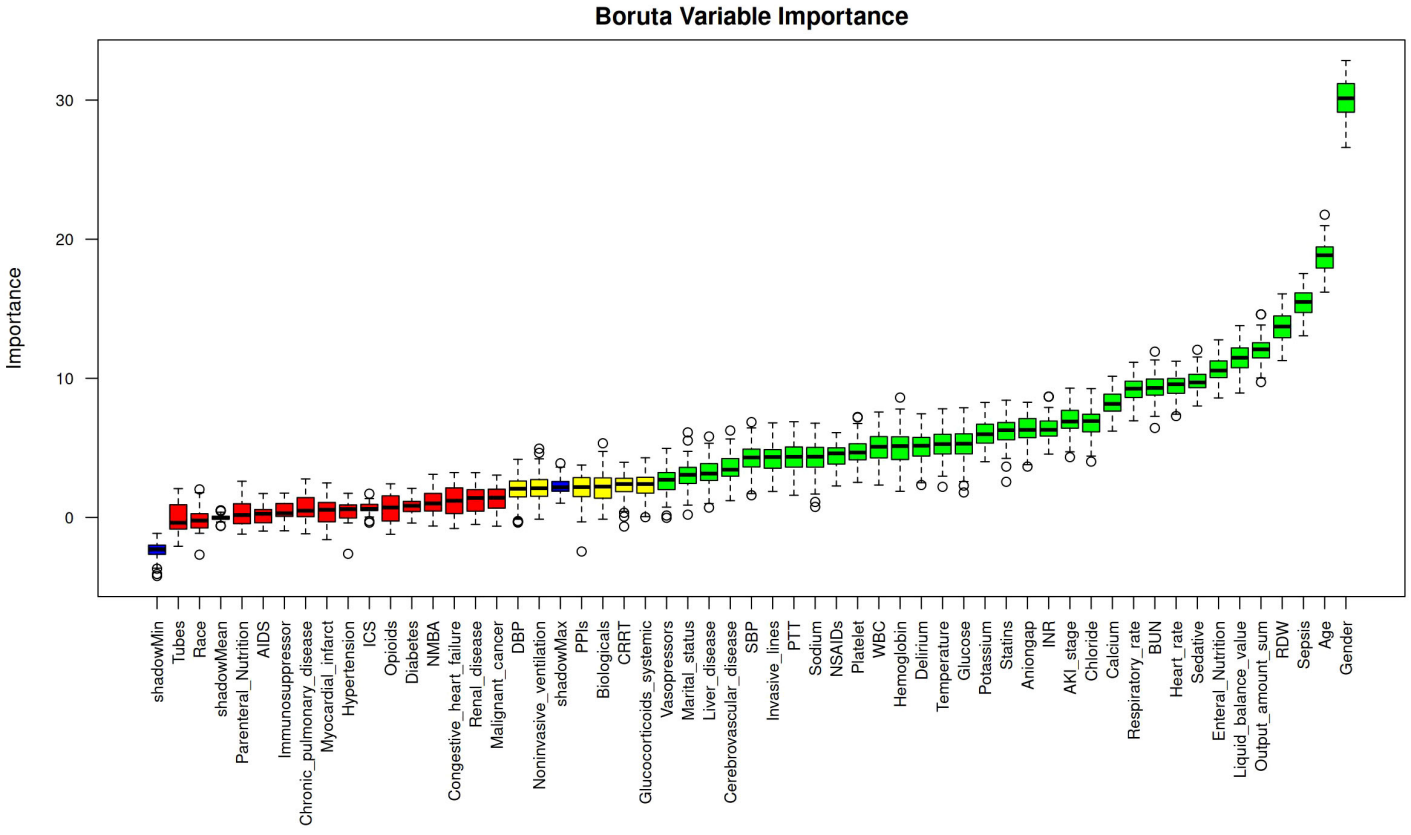
Variable importance ranking using the Boruta algorithm. Boxplots show the distribution of importance scores for each predictor variable assessed by the Boruta feature selection algorithm. Green boxplots indicate variables confirmed as important, red indicate variables rejected as unimportant, and yellow represent tentative variables. Blue boxplots correspond to shadow features created by Boruta for statistical comparison.

Feature selection was conducted using both the LASSO regression (**Figure 2**) and Boruta algorithm (**Figure 3**). A total of 28 variables were consistently identified by both Boruta and LASSO as robust predictors: Gender, Sepsis, Age, Sedative use, RDW, Enteral nutrition, Heart rate, Statins, Temperature, Glucocorticoids (systemic), Potassium, WBC, SBP, Anion gap, Calcium, Liver disease, Invasive lines, Respiratory rate, Liquid balance value, Delirium, BUN, NSAIDs, Sodium, Cerebrovascular disease, Noninvasive ventilation, PPIs, PTT, and CRRT. These intersecting variables were retained for subsequent model development.

### 3.3 Model performance

**Figure 4:**
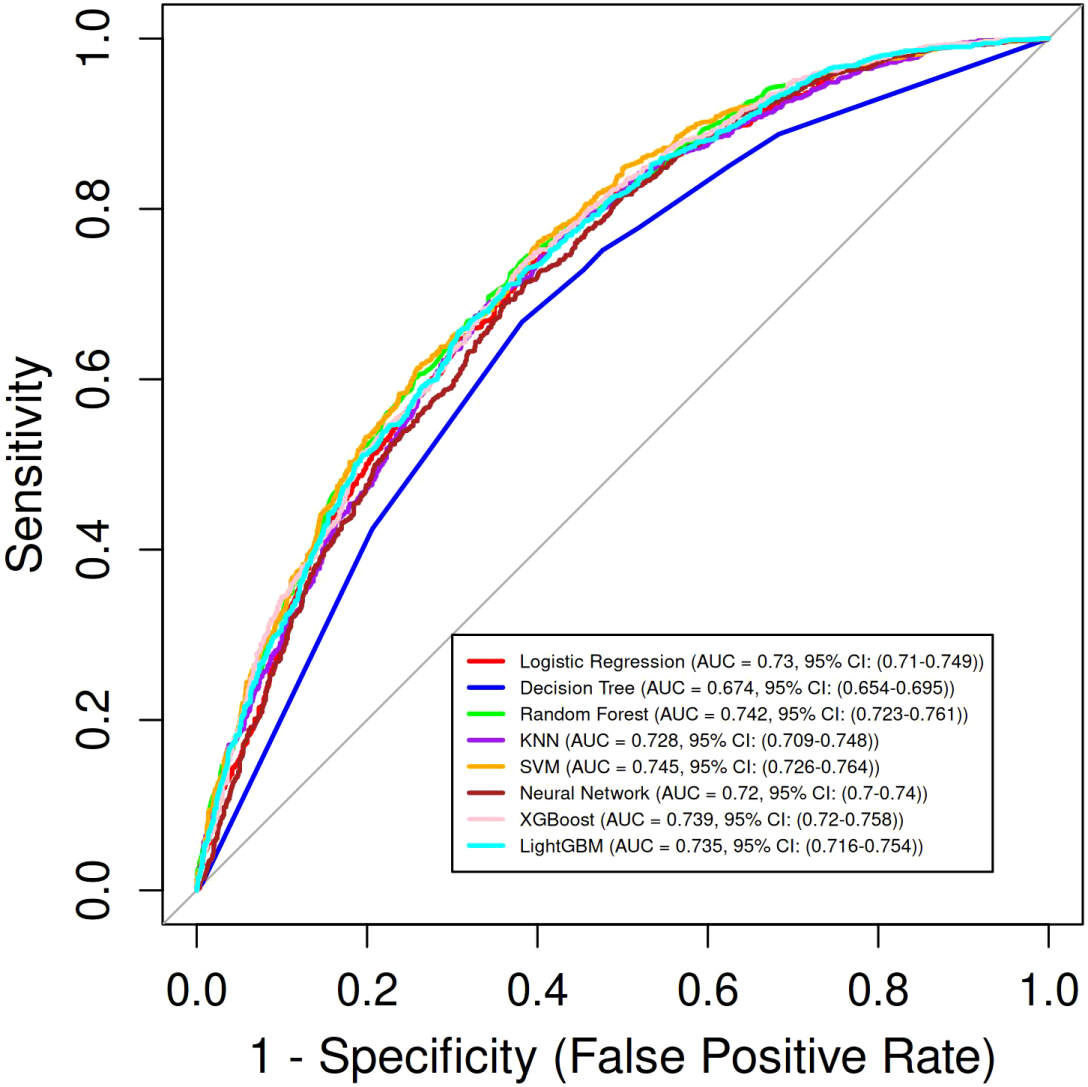
Receiver operating characteristic (ROC) curves for eight machine learning models in the validation cohort.

**Figure 5:**
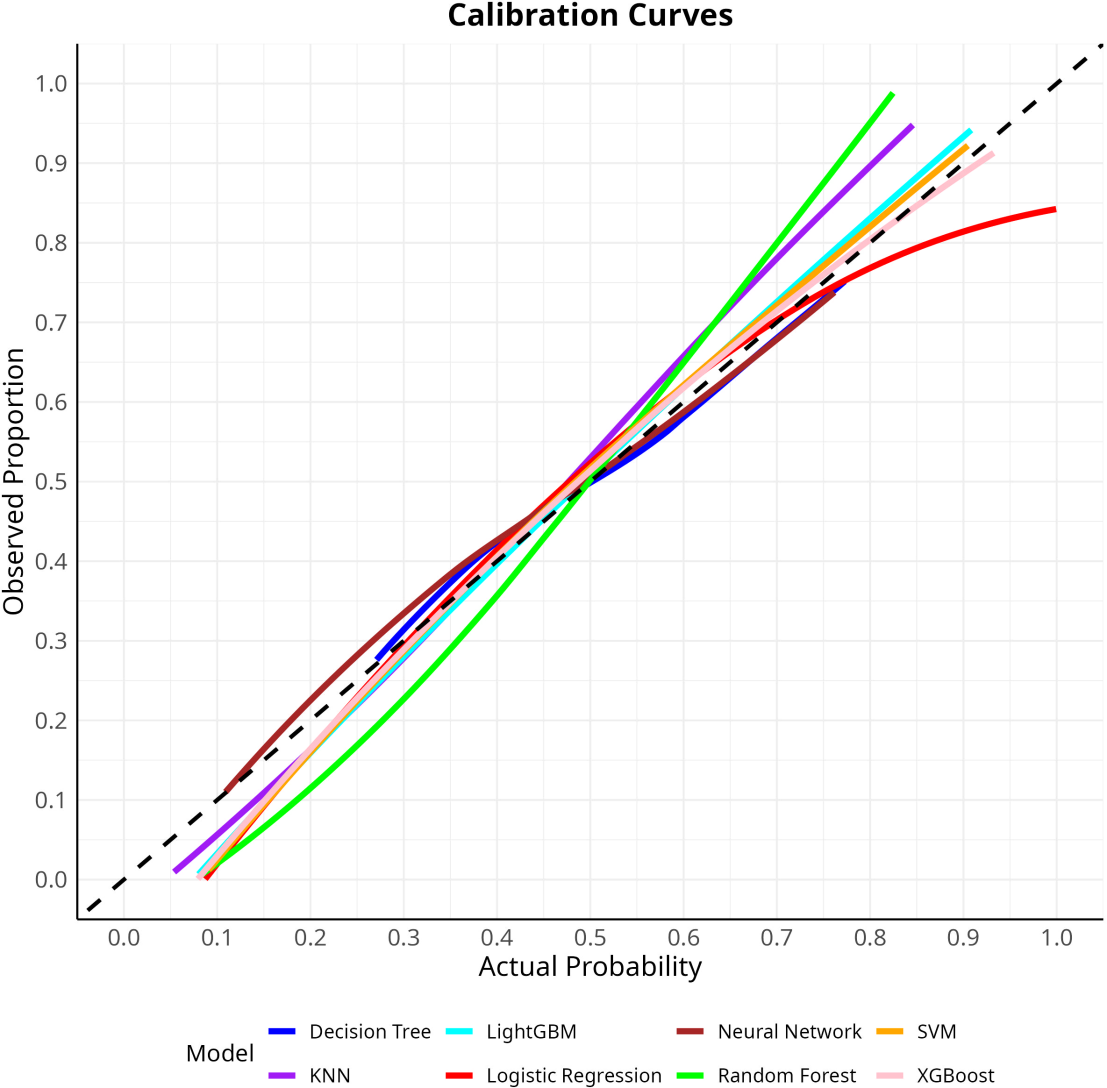
Calibration curves for eight machine learning models in the validation cohort.

**Figure 6:**
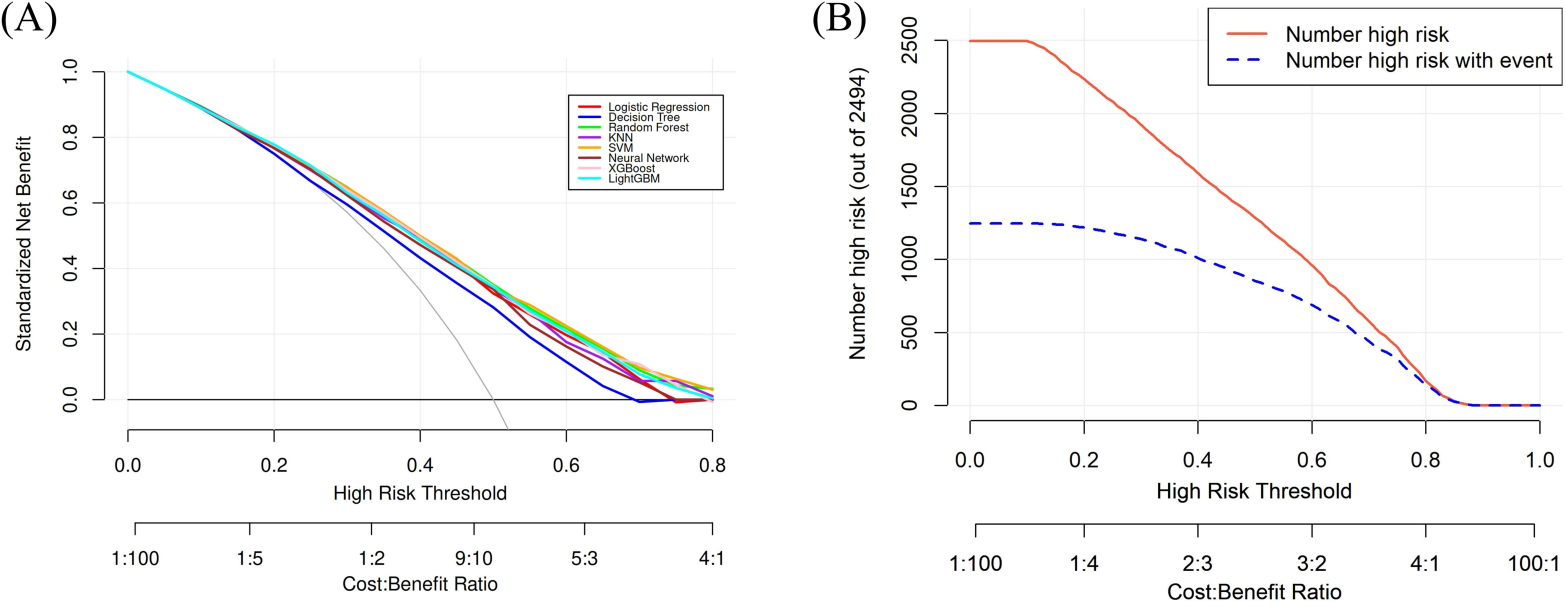
Decision curve analysis **(A)** for eight machine learning models and clinical impact curve **(B)** for the best prediction model (SVM) in the validation cohort. The solid black line in **(A)** represents the “treat-none” strategy (no patients are predicted as positive), which yields zero net benefit across all thresholds, and the dashed black line in **(A)** represents the “treat-all” strategy (all patients are predicted as positive), which corresponds to the expected net benefit if every patient were treated regardless of risk. Colored curves indicate the performance of each model, with higher curves suggesting greater clinical utility.

Eight machine learning models were constructed to predict the risk of *Escherichia coli* infection in ICU patients. Discriminative performance, as assessed by AUC, was shown in **Figure 4**. Among all models, the SVM achieved the highest AUC (0.745, 95% CI: 0.726-0.764), followed by RF (AUC = 0.742, 95% CI: 0.723-0.761) and XGBoost (AUC = 0.739, 95% CI: 0.720-0.758). The DT model yielded the lowest AUC (0.674, 95% CI: 0.654-0.695), while the remaining models, including LR, KNN, LightGBM, and NNet, demonstrated moderate discrimination (AUCs ranging from 0.719 to 0.739). In terms of sensitivity, the LightGBM and NNet models showed the highest values (0.711 and 0.703, respectively), whereas the KNN and LR models had the highest specificity (0.678 and 0.654, respectively). F1 scores were comparable across models (range: 0.662-0.688). Full details of performance metrics were provided in **Table 1**. Calibration curves for each model were displayed in **Figure 5**, demonstrating good agreement between predicted probabilities and observed outcomes. Decision curve analysis (**Figure 6A**) further confirmed the clinical utility of the models, with SVM, RF, and XGBoost consistently showing superior net benefit across a wide range of risk thresholds. Clinical impact curves of SVM (**Figure 6B**) indicated that, across a range of risk thresholds, the SVM model accurately identified a substantial number of patients at high risk for *E. coli* infection, with a considerable proportion of true positive cases among those classified as high risk. Overall, the SVM model exhibited the best balance between discrimination, calibration, and clinical utility for predicting *E. coli* infection in the validation cohort. We also explored the use of recursive feature elimination (RFE) for further variable reduction. However, as shown in **Supplementary Figure 3**, reducing the number of variables led to a notable decrease in model performance. Therefore, all 28 selected variables were retained for the final model development.

**Table 1:** Performance metrics of different machine learning models for predicting E. coli infection in the validation cohort.

Model	AUC	AUPRC	Sensitivity	Specificity	F1	Balanced accuracy
LR	0.730 (0.710-0.749)	0.696 (0.473-0.527)	0.672 (0.646-0.698)	0.654 (0.628-0.678)	0.666 (0.646-0.688)	0.663 (0.646-0.681)
KNN	0.728 (0.709-0.748)	0.704 (0.475-0.528)	0.654 (0.628-0.681)	0.678 (0.652-0.706)	0.662 (0.642-0.687)	0.666 (0.648-0.686)
DT	0.674 (0.654-0.695)	0.631 (0.474-0.527)	0.689 (0.663-0.714)	0.593 (0.565-0.620)	0.657 (0.636-0.678)	0.641 (0.622-0.659)
RF	0.742 (0.723-0.761)	0.721 (0.473-0.532)	0.712 (0.688-0.736)	0.636 (0.610-0.662)	0.686 (0.666-0.706)	0.674 (0.657-0.693)
XGBoost	0.739 (0.720-0.758)	0.710 (0.473-0.530)	0.702 (0.677-0.729)	0.644 (0.618-0.672)	0.683 (0.662-0.703)	0.673 (0.656-0.692)
LightGBM	0.735 (0.716-0.754)	0.710 (0.472-0.528)	0.711 (0.686-0.736)	0.634 (0.607-0.661)	0.685 (0.663-0.705)	0.672 (0.654-0.691)
SVM	0.745 (0.726-0.764)	0.723 (0.475-0.527)	0.682 (0.658-0.708)	0.657 (0.629-0.684)	0.673 (0.653-0.695)	0.669 (0.650-0.688)
NNet	0.720 (0.700-0.740)	0.678 (0.474-0.527)	0.588 (0.560-0.617)	0.708 (0.683-0.733)	0.625 (0.602-0.649)	0.648 (0.629-0.668)

LR, Logistic Regression; KNN, K-Nearest Neighbors; DT, Decision Tree; RF, Random Forest; XGBoost, Extreme Gradient Boosting; LightGBM, Light Gradient Boosting Machine; SVM, Support Vector Machine; NNet, Neural Network; AUC, area under the receiver operating characteristic curve; AUPRC, Area Under the Precision-Recall Curve; F1, F1 score.

### 3.4 Model interpretability with SHAP

**Figure 7:**
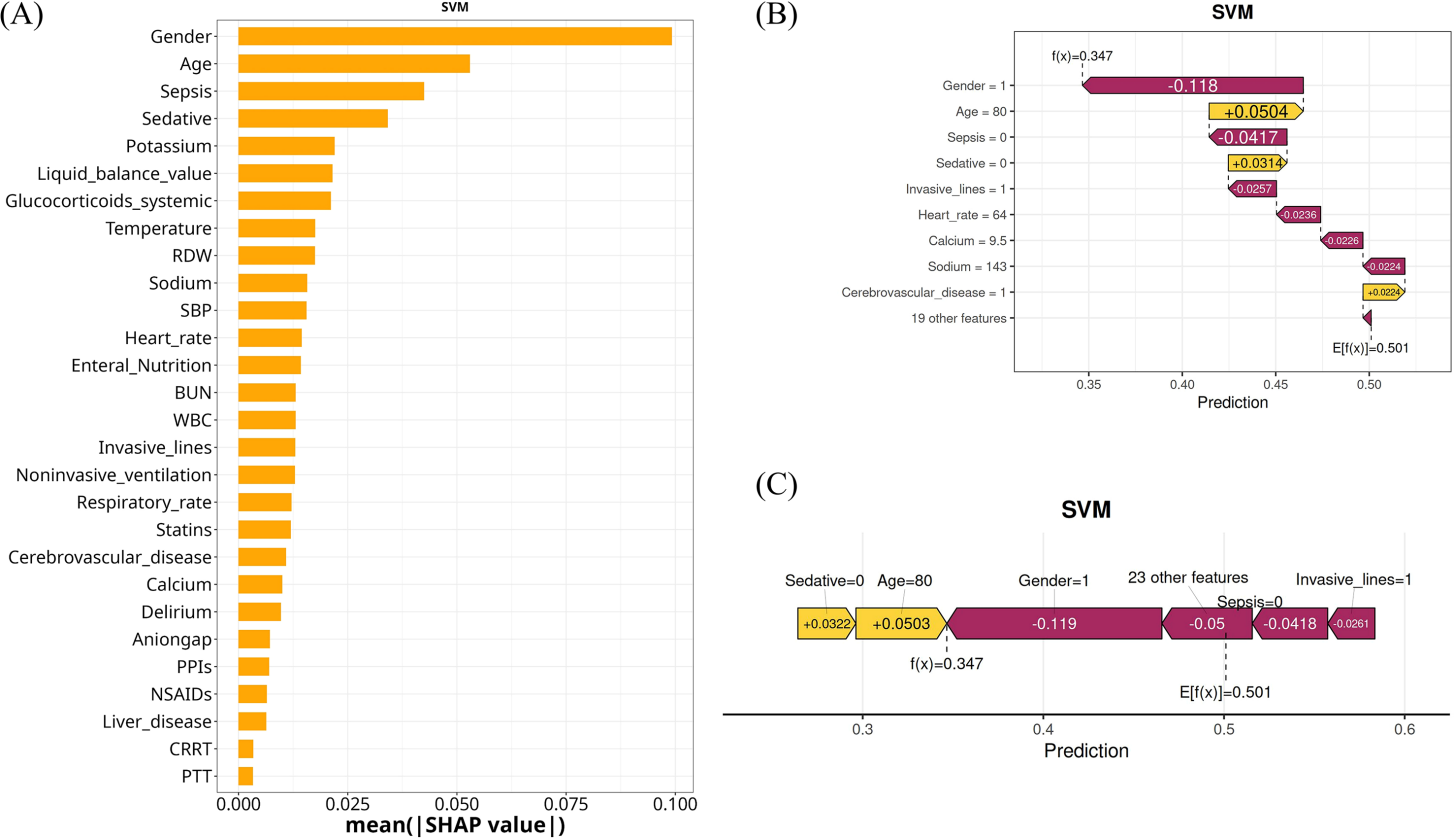
Interpretation of the SVM model using SHAP values. **(A)** Bar plot showing the mean absolute SHAP values for each variable. **(B)** SHAP force plot illustrating the impact of individual predictors on the model output for a representative patient. **(C)** SHAP decision plot visualizing the cumulative effect of key variables on the predicted outcome for a single case. SHAP, Shapley additive explanation.

To enhance interpretability and clinical credibility of the SVM model, we utilized SHAP to provide both global and local explanations for model predictions. Globally, the SHAP summary plot (**Figure 7A**) displayed the mean absolute SHAP value for each feature, ranking variables according to their overall contribution to *E. coli* infection prediction. Gender, Age, Sepsis, Sedative use, and Potassium were identified as the most influential predictors in the SVM model, followed by Liquid balance value, Glucocorticoids systemic use, Temperature, and RDW. These features had the largest impact on the model’s output, underlining their importance for clinical risk stratification. At the individual level, SHAP waterfall plot and force plot were used to decompose model predictions for specific patients (**Figures 7B, C**). For instance, in a representative patient, Gender=1 (male) and Age=80 substantially shifted the risk prediction, with additional contributions from Sedative use, Invasive lines, and other variables. Positive SHAP values indicated features that increased the predicted risk, while negative values indicated risk-reducing factors. We further explored the impact of each selected variable on the SVM model predictions by visualizing the relationship between feature values and their corresponding SHAP values. **Supplementary Figure 4** presented SHAP dependence plots for all 28 intersecting variables, ranked in descending order of importance. The SHAP decision process visually quantified how each variable contributed to the final probability, making the model’s reasoning transparent for clinicians.

### 3.5 Model application

**Figure 8:**
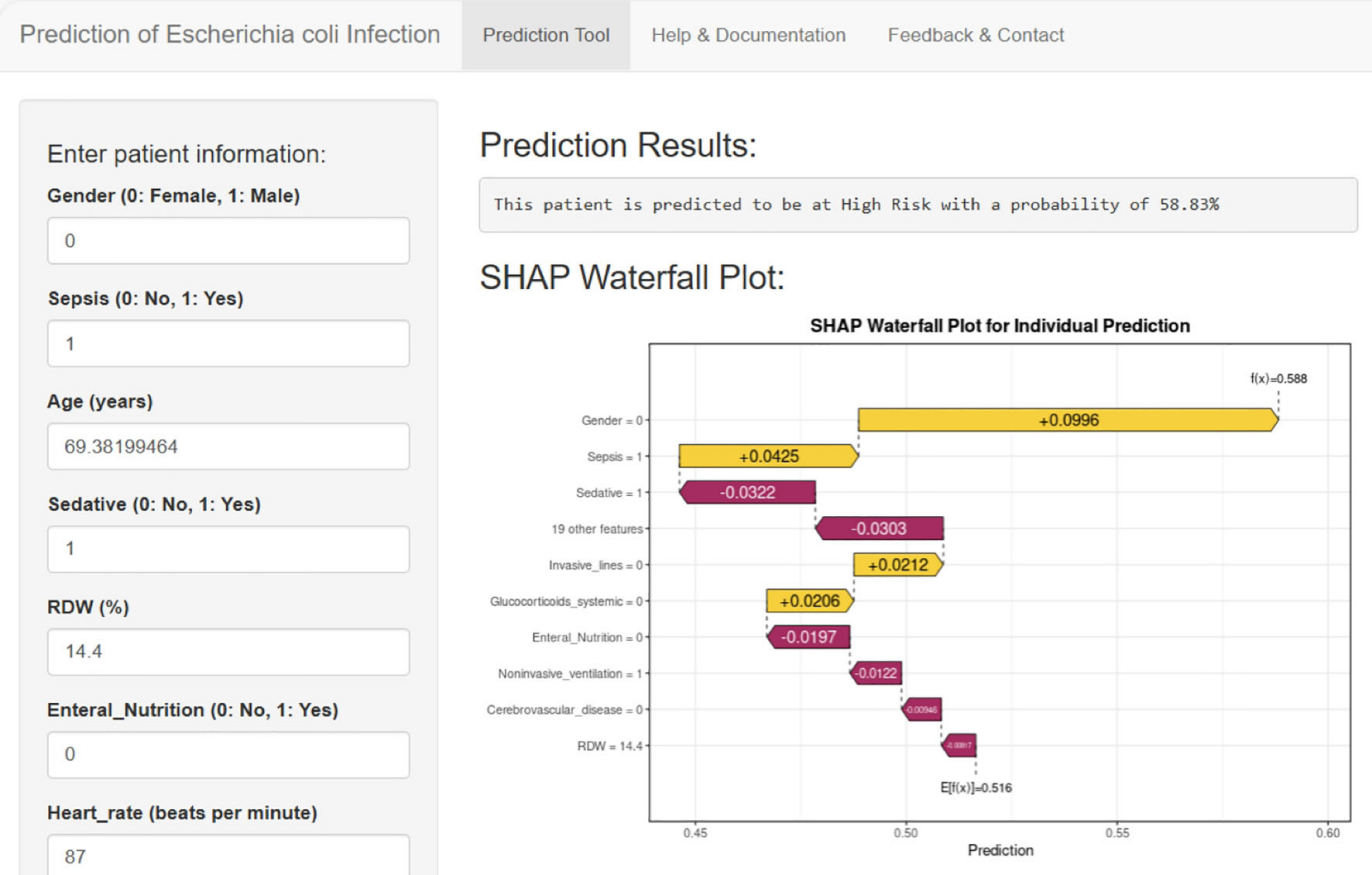
Web-based clinical decision support tool for individualized prediction of *E. coli* infection.

To enhance the clinical utility of our prediction model, we developed an online application based on the final SVM algorithm using the Shiny platform (**Figure 8**). This user-friendly tool enabled clinicians to rapidly assess the risk of *E. coli* infection by entering key patient variables available at ICU admission. Upon inputting patient data, the application could instantly calculate the individualized risk probability for *E. coli* infection and visually display the result in a straightforward format. Furthermore, to improve transparency and interpretability, the app provided a SHAP waterfall plot for each individual prediction, highlighting the contribution of each feature to the risk estimate. The online application can be visited through the website (Yang et al., 2025) (https://predicti.shinyapps.io/ecoli_predictor/).

## 4 Discussion

In this study, we developed and validated a suite of machine learning models for individualized risk prediction of *Escherichia coli* infection among ICU patients. The SVM model demonstrated the best overall performance, achieving robust discrimination and calibration in the validation cohort. The use of SHAP analysis enhanced interpretability, enabling both global and individualized insights into feature contributions and model decision-making. Furthermore, our development of an online risk calculator provides a practical tool for real-time clinical implementation.

While machine learning methods have been increasingly applied in infection prediction and risk stratification, there remains a notable absence of validated models specifically targeting the risk of *Escherichia coli* infection in ICU populations. Previous studies have primarily addressed risk factors for infection (Qi et al., 2024) or focused on antimicrobial resistance (Yu et al., 2018; Xu et al., 2025), but not on *E. coli* infection risk prediction in the intensive care setting. Our study addressed this critical gap by providing a validated and interpretable tool for individualized risk stratification in this high-risk population. In contrast to previous work such as Clarke et al., which leveraged extensive historical health records and outpatient data to predict invasive *E. coli* disease in a general adult population (Clarke et al., 2024), our approach was tailored to the ICU context. We focused on variables available at ICU admission, emphasizing acute clinical, laboratory, and treatment characteristics, as ICU patients differed markedly from the general population in terms of disease acuity, clinical complexity, and risk exposure. By centering on immediately available and actionable data, our model was well suited for real-time risk assessment and clinical decision-making in critically ill patients. Notably, both our study and that of Clarke et al. identified advanced age and female sex as key risk factors, consistently demonstrating that older adults, particularly women, are at substantially increased risk. This heightened susceptibility is attributable to a combination of physiological, anatomical, and clinical factors. Aging is associated with immunosenescence, which impairs the host’s ability to mount an effective immune response against bacterial pathogens, as well as reduced organ reserve that limits resilience to acute stressors. Older adults often present with multiple chronic comorbidities, such as diabetes and chronic kidney disease, further predisposing them to infection (Fuchs et al., 2012; Brunker et al., 2023). In the ICU setting, elderly patients are more likely to undergo invasive procedures like urinary catheterization and experience prolonged hospitalization, both of which increase the opportunity for nosocomial *E. coli* infection. Additionally, frequent exposure to broad-spectrum antibiotics in this population promotes colonization with resistant organisms. Female sex confers added vulnerability, primarily due to anatomical differences. In our study, the majority of *Escherichia coli* infections in ICU patients originated from the urinary tract, as illustrated in **Supplementary Figure 5**. The female urethra is shorter and in closer proximity to the perianal area, facilitating the ascent of enteric bacteria such as *E. coli* into the urinary tract. Hormonal changes, particularly reduced estrogen levels in postmenopausal women, may further compromise mucosal defenses. Under critical illness, these age- and sex-related risks are magnified, underscoring the importance of targeted infection prevention and surveillance strategies in older female ICU patients.

Sepsis significantly increased the risk of secondary infections such as *Escherichia coli* among ICU patients. Sepsis was associated with dysfunction of the intestinal barrier, resulting in increased permeability that facilitated bacterial translocation from the gut into the systemic circulation (Haussner et al., 2019). Recent studies have demonstrated that, following sepsis and broad-spectrum antibiotic therapy, there was a marked reduction in gut microbiota diversity, with enrichment of antibiotic-resistant bacteria such as *E. coli*. Genomic analyses have shown a high degree of homology between gut-dominant bacteria and pathogens isolated from secondary infection sites, indicating that intestinal colonization was a major source of subsequent systemic infection in septic patients (Mu et al., 2022). Furthermore, sepsis induced profound immune dysregulation, including the reprogramming of granulocytes to a hyper-inflammatory yet functionally compromised state, which paradoxically impaired effective clearance of new infections and increased susceptibility to opportunistic pathogens (Wang et al., 2023). Collectively, these alterations in mucosal barrier integrity, microbiome composition, and immune function converged to create a highly permissive environment for secondary *E. coli* infections in the ICU setting.

Previous studies have consistently shown that the use of sedative agents, particularly benzodiazepines, in critically ill patients was associated with an increased risk of healthcare-associated infections, including ventilator-associated pneumonia and bloodstream infections (Caroff et al., 2016). This elevated risk was believed to result from the suppression of protective airway reflexes, impaired gastrointestinal and urinary tract motility, and the immunomodulatory effects of sedatives, especially when used at greater depth or duration. However, in our study, the use of sedative medications was paradoxically associated with a decreased risk of *Escherichia coli* infection. Several factors may explain this counterintuitive finding. First, sedated patients often receive more intensive nursing care, including rigorous infection prevention measures and frequent monitoring, which may reduce the risk of nosocomial infections. Second, in modern ICU practice, the indication for sedation often correlates with early invasive management, such as mechanical ventilation and the use of indwelling devices. However, current care bundles prioritize prompt weaning and timely removal of invasive devices whenever possible. This strategy shortens the duration of exposure to potential infection sources, such as urinary or vascular catheters, thus potentially reducing the risk of device-associated infections, including urinary tract colonization. Interestingly, there is also some *in vitro* evidence that certain sedatives, such as midazolam, possessed direct antimicrobial activity against *E. coli* and other clinically relevant pathogens, while other agents like propofol and dexmedetomidine do not exhibit such effects (Keleş et al., 2006). Lastly, it is possible that our machine learning model may have captured correlations influenced by clinical practices unique to our cohort, rather than direct causal effects. These findings underscore the complexity of infection risk stratification in ICU populations and highlight the need for further large-scale, prospective studies to clarify the relationship between sedative use and pathogen-specific infection risk.

The clinical application of our predictive model provides actionable insights for infection prevention in ICU patients. By highlighting the most influential variables, such as female sex, advanced age, and sepsis, our model enables clinicians to identify patients at particularly high risk for *Escherichia coli* infection. For these individuals, interventions should include heightened surveillance and prompt management of modifiable risk factors. Strict adherence to aseptic technique, frequent assessment of catheter necessity, and timely removal are essential to reduce urinary tract infection risk. For prevention of extra-urinary *E. coli* infections, such as respiratory or bloodstream infections, measures include minimizing the duration of mechanical ventilation, employing subglottic suctioning, ensuring proper oral care, and maintaining strict asepsis for vascular lines with early removal when feasible. Meticulous daily hygiene care, early mobilization, and nutritional and immune support can further enhance host defenses across infection sites. Abnormal electrolyte levels and positive fluid balance should be corrected promptly. Cautious use of sedatives is warranted, with preference for light sedation and regular reassessment to avoid unnecessary exposure and associated complications. Importantly, our findings underscore the need for a personalized and comprehensive approach, that integrating risk predictions into clinical workflows allows for tailored prevention, targeted device management, and broad infectious surveillance, ultimately reducing the burden and adverse outcomes of *E. coli* infection in vulnerable ICU populations.

This study has several limitations. First, although our model achieved an acceptable level of discrimination, the AUC was relatively modest compared with some previously published infection prediction models. This may be partly attributable to the increased difficulty of predicting infection by a specific pathogen (*E. coli*) at an early stage, rather than broader categories such as hospital-acquired or multidrug-resistant infections. Moreover, the predictors used in this study were limited to those available at or shortly after ICU admission to ensure timeliness of prediction. While this design enhances the model’s clinical applicability for early warning, it inevitably restricts the number and depth of predictive features, which may constrain performance. Second, this work was based on a single-center retrospective dataset, which may limit the generalizability of our findings across different institutions and patient populations. Another methodological limitation lies in the handling of class imbalance. Although *E. coli* infection accounted for only 7.9% of the cohort, the absolute number of positive cases (n = 4, 157) was sufficiently large to support robust model training even after undersampling. We selected undersampling as the primary balancing strategy because it preserves the authenticity of real-world data and avoids introducing synthetic samples that may generate artificial patterns or amplify noise. This approach has also been adopted in recent ICU-based machine learning studies with similar imbalance ratios (Guan et al., 2024). However, undersampling inevitably reduces the number of negative cases used for training, which may lead to information loss and limit the model’s ability to capture rare but clinically relevant patterns in the majority class. Compared with oversampling methods such as SMOTE (Alkhawaldeh et al., 2023), undersampling provides a simpler and more conservative approach that reduces computational burden and the risk of overfitting from synthetic data, but at the expense of potential data inefficiency. Alternative resampling or hybrid approaches, including SMOTE-Tomek or adaptive ensemble methods, may help achieve a better trade-off between data integrity and model performance, and will be considered in future research. Additionally, although k-fold cross-validation and an independent validation set were employed to mitigate overfitting, the possibility of overfitting cannot be completely excluded. Finally, while SHAP analysis improves model interpretability, it cannot establish causal relationships, and some observed associations may reflect residual confounding or institution-specific practices.

Future research should focus on several directions to further strengthen and extend this work. First, multi-center and prospective validation is needed to evaluate the model’s generalizability and robustness across different healthcare systems, patient populations, and clinical environments. Second, incorporating more granular and longitudinal data, such as serial physiological measurements, detailed microbiological results, prior infection history, and antibiotic exposure, may enhance predictive accuracy and enable dynamic risk monitoring during ICU stay. In addition, employing more advanced hyperparameter-tuning approaches, such as Bayesian optimization, randomized search, or genetic algorithms, together with ensemble-learning frameworks (e.g., stacking or blending multiple base learners), may further refine model calibration and stability. Furthermore, as the size and diversity of available ICU datasets continue to grow, deep learning approaches, such as artificial neural networks (ANNs), convolutional neural networks (CNNs), and recurrent neural networks (RNNs), could be leveraged to automatically learn high-order nonlinear relationships among complex clinical and microbiological variables. These architectures may provide enhanced representational capacity and predictive performance compared with traditional machine learning algorithms, while techniques such as attention mechanisms and integrated gradient analysis could further improve interpretability. Finally, interventional and implementation studies are warranted to determine whether integrating this prediction model into routine ICU workflows can effectively support early identification of high-risk patients, improve preventive decision-making, and ultimately reduce the incidence and adverse outcomes of E. coli infections.

## 5 Conclusions

In summary, our study demonstrates that machine learning-based prediction models can provide accurate, interpretable, and actionable risk assessment for *E. coli* infection in ICU patients. The deployment of such tools in clinical practice has the potential to improve infection prevention, patient outcomes, and healthcare resource utilization in the intensive care setting.

## Data Availability

Publicly available datasets were analyzed in this study. This data can be found here: https://physionet.org/content/mimiciv/3.1/.

## Glossary

AIDS: Acquired Immune Deficiency Syndrome
AKI: Acute Kidney Injury
ALP: Alkaline Phosphatase
ALT: Alanine Aminotransferase
AMR: Antimicrobial Resistance
ANNs: Artificial Neural Networks
APS III: Acute Physiology Score III
AST: Aspartate Aminotransferase
AUC: Area Under the Curve
AUPRC: Area Under the Precision-Recall Curve
BUN: Blood Urea Nitrogen
CI: Confidence Interval
CIC: Clinical Impact Curve
CITI: Collaborative Institutional Training Initiative
CNNs: Convolutional Neural Networks
CRP: C-Reactive Protein
CRRT: Continuous Renal Replacement Therapy
DCA: Decision Curve Analysis
DBP: Diastolic Blood Pressure
DT: Decision Tree
E. coli: Escherichia coli
GCS: Glasgow Coma Scale
GGT: Gamma-Glutamyl Transferase
ICD: International Classification of Diseases
ICS: Inhaled Corticosteroids
ICU: Intensive Care Unit
INR: International Normalized Ratio
IQR: Interquartile Range
KNN: K-Nearest Neighbors
LASSO: Least Absolute Shrinkage and Selection Operator
LDH: Lactate Dehydrogenase
LightGBM: Light Gradient Boosting Machine
LODS: Logistic Organ Dysfunction Score
MIMIC-IV: Medical Information Mart for Intensive Care IV
MIT: Massachusetts Institute of Technology
NNet: Neural Network
NMBA: Neuromuscular Blocking Agents
NSAIDs: Nonsteroidal Anti-inflammatory Drugs
OASIS: Oxford Acute Severity of Illness Score
PaO₂/FiO₂: Ratio of Arterial Oxygen Partial Pressure to Fraction of Inspired Oxygen
PCO₂: Partial Pressure of Carbon Dioxide
PF: Pulmonary Fibrosis
PO₂: Partial Pressure of Oxygen
PPIs: Proton Pump Inhibitors
PT: Prothrombin Time
PTT: Partial Thromboplastin Time
RBC: Red Blood Cell
RDW: Red Cell Distribution Width
RF: Random Forest
RNNs: Recurrent Neural Networks
ROC: Receiver Operating Characteristic
SAPS II: Simplified Acute Physiology Score II
SBP: Systolic Blood Pressure
SD: Standard Deviation
SHAP: Shapley Additive Explanations
SIRS: Systemic Inflammatory Response Syndrome
SMOTE: Synthetic Minority Over-Sampling Technique
SO₂: Oxygen Saturation
SOFA: Sequential Organ Failure Assessment
SQL: Structured Query Language
SVM: Support Vector Machine
WBC: White Blood Cell
XGBoost: Extreme Gradient Boosting.
