# Supplementary material for: An interpretable machine learning model for early prediction of Escherichia coli infection in ICU patients
Source: Front Cell Infect Microbiol. 2025 Nov 24;15:1682764. doi: 10.3389/fcimb.2025.1682764 (PMC12682895; doi:10.3389/fcimb.2025.1682764)
Supplement: Supplementary file 1 [file Table1.docx]

**Supplementary Table 1** Details of missing data.

| Variable | Missing Count | Missing Ratio |
| --- | --- | --- |
| Age | 0 | 0 |
| Gender | 0 | 0 |
| Race | 0 | 0 |
| Marital status | 0 | 0 |
| Weight | 22266 | 0.423679 |
| Height | 51502 | 0.979982 |
| Input amount sum | 5546 | 0.10553 |
| Output amount sum | 999 | 0.019009 |
| Liquid balance value | 6269 | 0.119287 |
| Urine output sum | 1273 | 0.024223 |
| Heart rate | 1 | 1.90E-05 |
| SBP | 3 | 5.71E-05 |
| DBP | 3 | 5.71E-05 |
| Respiratory rate | 43 | 0.000818 |
| Temperature | 204 | 0.003882 |
| WBC | 437 | 0.008315 |
| Neutrophils abs | 28179 | 0.536191 |
| Monocytes abs | 28179 | 0.536191 |
| Lymphocytes abs | 28160 | 0.53583 |
| Eosinophils abs | 28177 | 0.536153 |
| Basophils abs | 28179 | 0.536191 |
| Neutrophils | 28163 | 0.535887 |
| Monocytes | 28163 | 0.535887 |
| Lymphocytes | 28144 | 0.535525 |
| Eosinophils | 28164 | 0.535906 |
| Basophils | 28163 | 0.535887 |
| CRP | 50174 | 0.954713 |
| RBC | 435 | 0.008277 |
| Hemoglobin | 439 | 0.008353 |
| Hematocrit | 407 | 0.007744 |
| RDW | 448 | 0.008525 |
| Platelet | 431 | 0.008201 |
| Albumin | 32151 | 0.611771 |
| GGT | 51882 | 0.987213 |
| ALT | 25075 | 0.477128 |
| ALP | 25277 | 0.480972 |
| AST | 24884 | 0.473494 |
| Bilirubin total | 25211 | 0.479716 |
| BUN | 390 | 0.007421 |
| Creatinine | 384 | 0.007307 |
| LDH | 32458 | 0.617612 |
| Calcium | 2748 | 0.052289 |
| Potassium | 392 | 0.007459 |
| Sodium | 380 | 0.007231 |
| Glucose | 469 | 0.008924 |
| Chloride | 380 | 0.007231 |
| Anion gap | 413 | 0.007859 |
| D dimer | 51812 | 0.985881 |
| Fibrinogen | 35977 | 0.684572 |
| INR | 5027 | 0.095654 |
| PT | 5027 | 0.095654 |
| PTT | 5241 | 0.099726 |
| PO2 | 18540 | 0.35278 |
| PCO2 | 18544 | 0.352856 |
| PaO2 FiO2 ratio | 26269 | 0.499848 |
| SO2 | 35126 | 0.668379 |
| Lactate | 22104 | 0.420596 |
| PH | 18544 | 0.352856 |
| Bicarbonate | 51168 | 0.973627 |
| Base excess | 18544 | 0.352856 |
| Myocardial infarct | 0 | 0 |
| Congestive heart failure | 0 | 0 |
| Cerebrovascular disease | 0 | 0 |
| Chronic pulmonary disease | 0 | 0 |
| Liver disease | 0 | 0 |
| Renal disease | 0 | 0 |
| Diabetes | 0 | 0 |
| Hypertension | 0 | 0 |
| Malignant cancer | 0 | 0 |
| AIDS | 0 | 0 |
| AKI | 0 | 0 |
| AKI stage | 0 | 0 |
| Sepsis | 0 | 0 |
| Delirium | 0 | 0 |
| Glucocorticoids systemic | 0 | 0 |
| ICS | 0 | 0 |
| Immunosuppressor | 0 | 0 |
| Biologicals | 0 | 0 |
| Vasopressors | 0 | 0 |
| PPIs | 0 | 0 |
| NMBA | 0 | 0 |
| Sedative | 0 | 0 |
| Opioids | 0 | 0 |
| NSAIDs | 0 | 0 |
| Statins | 0 | 0 |
| Invasive ventilation | 0 | 0 |
| Noninvasive ventilation | 0 | 0 |
| CRRT | 0 | 0 |
| Invasive lines | 0 | 0 |
| Tubes | 0 | 0 |
| Enteral Nutrition | 0 | 0 |
| Parenteral Nutrition | 0 | 0 |
| antibiotic | 0 | 0 |
| Invasive ventilation hours | 0 | 0 |
| Noninvasive ventilation hours | 0 | 0 |
| CRRT days | 0 | 0 |
| APSIII | 0 | 0 |
| LODS | 0 | 0 |
| OASIS | 0 | 0 |
| SIRS | 0 | 0 |
| SOFA | 68 | 0.001294 |
| SAPSII | 0 | 0 |
| GCS | 91 | 0.001732 |
| Charlson comorbidity index | 0 | 0 |
| Los hospital | 0 | 0 |
| Hospital mortality | 0 | 0 |
| ICU time 14d | 0 | 0 |
| ICU outcome 14d | 0 | 0 |
| ICU time 28d | 0 | 0 |
| ICU outcome 28d | 0 | 0 |
| ICU time 30d | 0 | 0 |
| ICU outcome 30d | 0 | 0 |
| ICU time 90d | 0 | 0 |
| ICU outcome 90d | 0 | 0 |
| ICU time 365d | 0 | 0 |
| ICU outcome 365d | 0 | 0 |

Abbreviation: SBP, systolic blood pressure; DBP, diastolic blood pressure; WBC, white blood cell; CRP, C-reactive protein; RBC, red blood cell; RDW, red cell distribution width; GGT, gamma-glutamyl transferase; ALT, alanine aminotransferase; ALP, alkaline phosphatase; AST, aspartate aminotransferase; BUN, blood urea nitrogen; LDH, lactate dehydrogenase; INR, international normalized ratio; PT, prothrombin time; PTT, partial thromboplastin time; PO₂, partial pressure of oxygen; PCO₂, partial pressure of carbon dioxide; PaO₂/FiO₂ ratio, partial pressure of oxygen to fraction of inspired oxygen ratio; SO₂, oxygen saturation; AKI, acute kidney injury; ICS, inhaled corticosteroids; PPIs, proton pump inhibitors; NMBA, neuromuscular blocking agents; NSAIDs, non-steroidal anti-inflammatory drugs; CRRT, continuous renal replacement therapy; APSIII, Acute Physiology Score III; LODS, Logistic Organ Dysfunction Score; OASIS, Oxford Acute Severity of Illness Score; SIRS, systemic inflammatory response syndrome; SOFA, Sequential Organ Failure Assessment; SAPSII, Simplified Acute Physiology Score II; GCS, Glasgow Coma Scale; LOS, length of stay.


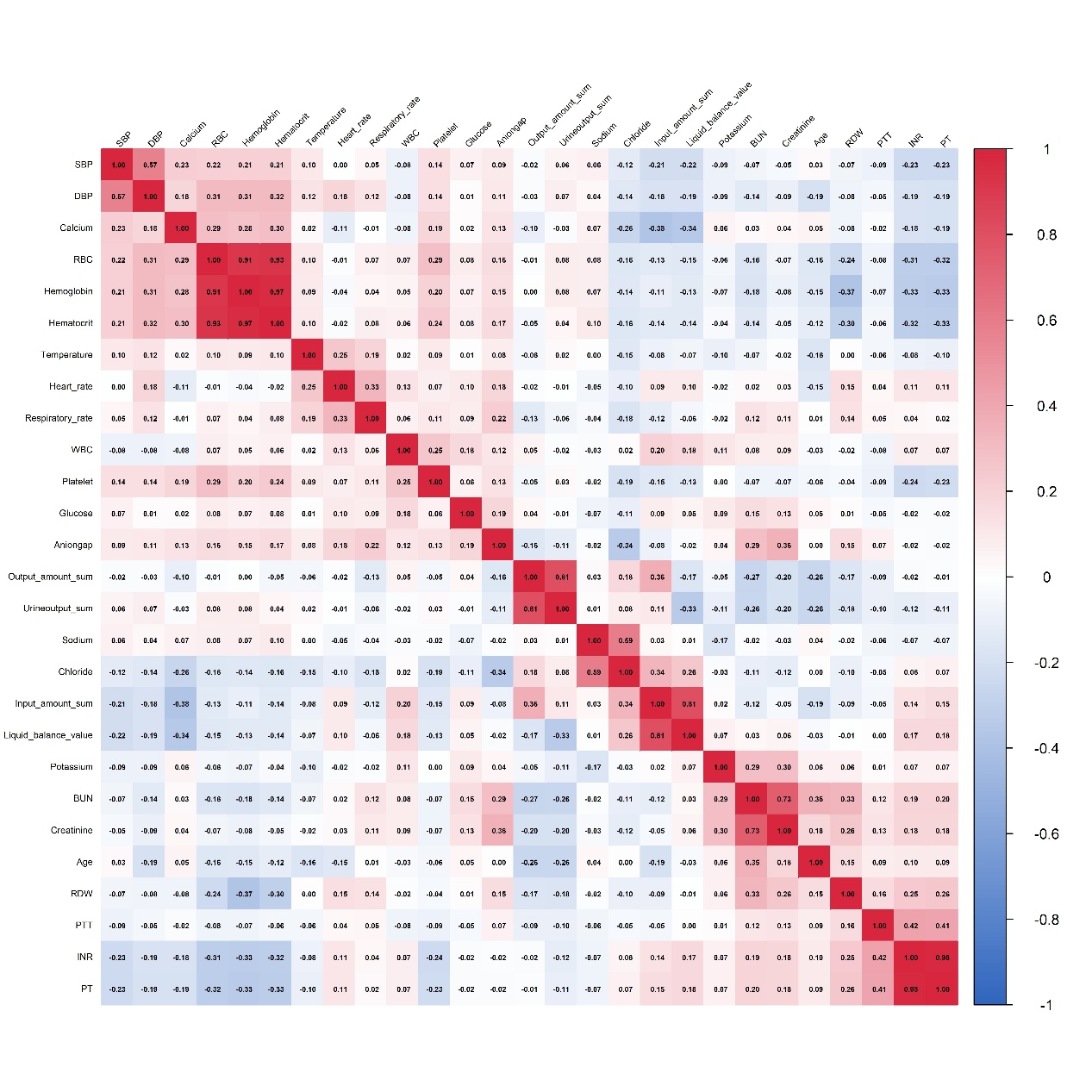


**Supplementary Figure 1** The heatmap-based Spearman Correlation Analysis. Abbreviations: SBP, systolic blood pressure; DBP, diastolic blood pressure; RBC, red blood cell count; WBC, white blood cell count; BUN, blood urea nitrogen; RDW, red cell distribution width; PTT, partial thromboplastin time; INR, international normalized ratio; PT, prothrombin time.


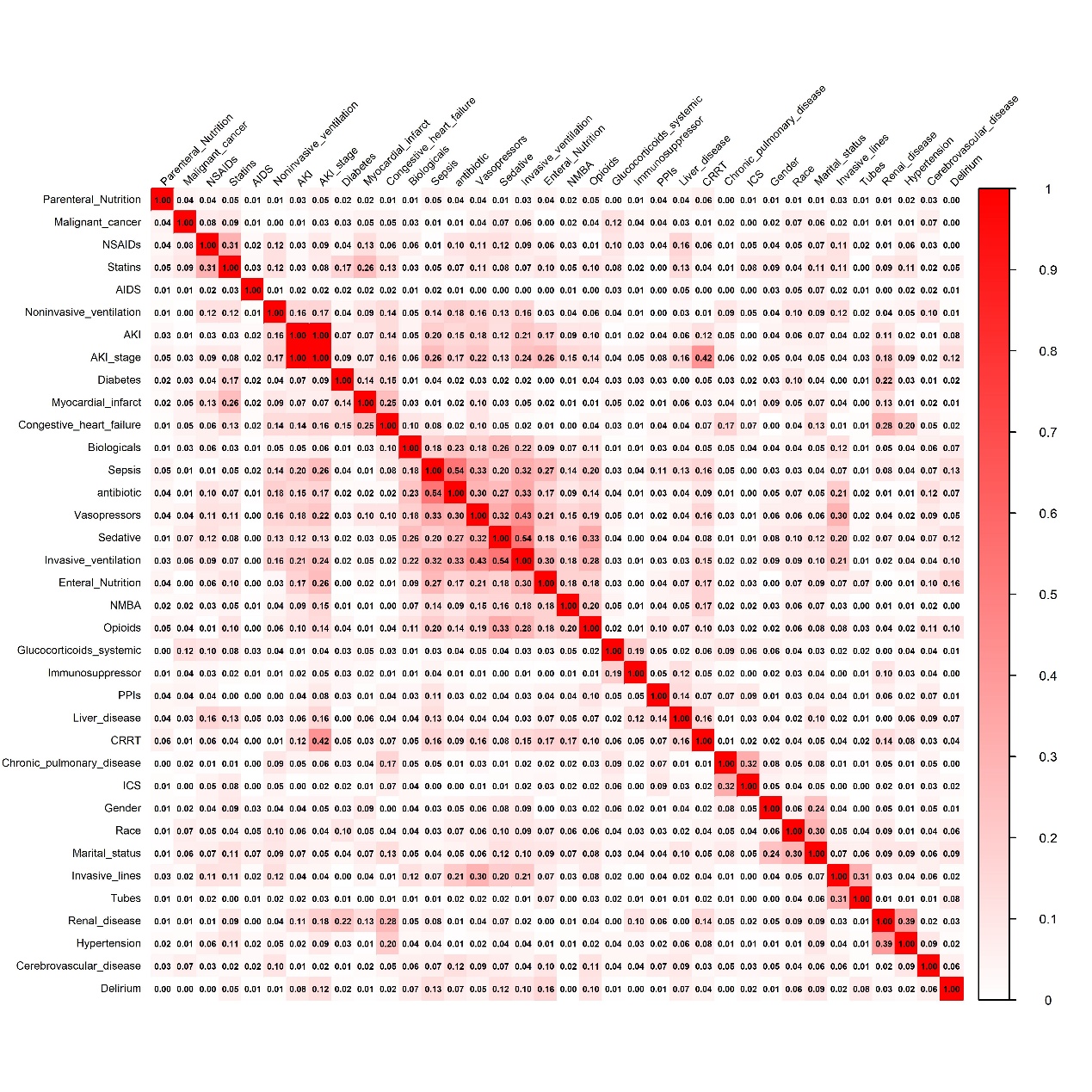


**Supplementary Figure 2** The heatmap-based Cramer’ V test. Abbreviations: NSAIDs, nonsteroidal anti-inflammatory drugs; AKI, acute kidney injury; NMBA, neuromuscular blocking agents; PPIs, proton pump inhibitors; CRRT, continuous renal replacement therapy; ICS, inhaled corticosteroids.

**Supplementary Table 2** Optimized Hyperparameters and Performance of Machine Learning Models.

| Model | Hyperparameter Search Range | Optimal Parameters |
| --- | --- | --- |
| LR | — | Default logistic (binomial) |
| KNN | k = 30–210 (step = 5);  kernel = triangular;  distance = 1 | k = 170;  kernel = triangular;  distance = 1 |
| DT | cp = 0.0001–0.01;  maxdepth = 6–10 | cp = 0.0036;  maxdepth = 6 |
| RF | mtry = 1–√p;  ntree = 50–700 | mtry = 2;  ntree = 600 |
| XGBoost | max_depth = 3–10;  η = 0.01–0.3;  nrounds = 50–150 | max_depth = 3;  η = 0.1;  nrounds = 100 |
| LightGBM | num_leaves = (15,31);  max_depth = −1–3;  learning_rate = 0.1–0.2;  λ₁/λ₂ = 0–1 | num_leaves = 15;  max_depth = −1;  learning_rate = 0.1;  λ₁ = 0; λ₂ = 0 |
| SVM | C = 10⁻¹–10³;  σ = 10⁻³–10¹ | C = 10;  σ = 0.01 |
| NNet | Hidden layers: (2), (3), (4), (5), (2,1), (2,2) | hidden = (2,2) |

Abbreviations: LR, Logistic Regression; KNN, K-Nearest Neighbors; DT, Decision Tree; RF, Random Forest; XGBoost, Extreme Gradient Boosting; LightGBM, Light Gradient Boosting Machine; SVM, Support Vector Machine; NNet, Neural Network.

**Supplementary Table 3** Baseline characteristics of patients in non-*E. coli* group and *E. coli* group.

| Variables | non-E. coli (n=48397) | E. coli(n=4157) | *P* value |
| --- | --- | --- | --- |
| Age, years | 65.06 (16.71) | 70.10 (15.80) | <.001 |
| Gender (%) | 28307 (58.5) | 1498 (36.0) | <.001 |
| Race (%) |  |  | .106 |
| White | 31818 (65.7) | 2768 (66.6) |  |
| Black | 4281 (8.8) | 373 (9.0) |  |
| Yellow | 1436 (3.0) | 141 (3.4) |  |
| Other | 10862 (22.4) | 875 (21.0) |  |
| Marital status (%) |  |  | <.001 |
| Single | 12566 (26.0) | 1053 (25.3) |  |
| Married | 22089 (45.6) | 1666 (40.1) |  |
| Divorced | 3337 (6.9) | 326 (7.8) |  |
| widowed | 5325 (11.0) | 711 (17.1) |  |
| Unknown | 5080 (10.5) | 401 (9.6) |  |
| Input amount sum, ml | 4357.89 (3113.02) | 4485.84 (3700.74) | .012 |
| Output amount sum, ml | 2597.17 (2619.90) | 2274.29 (4367.53) | <.001 |
| Liquid balance value, ml | 1773.42 (3468.66) | 2210.80 (5323.45) | <.001 |
| Urineoutput sum, ml | 1861.54 (1234.03) | 1596.89 (1147.74) | <.001 |
| Heart rate, ml | 87.32 (19.84) | 91.19 (20.58) | <.001 |
| SBP, mmHg | 124.77 (24.32) | 122.84 (25.19) | <.001 |
| DBP, mmHg | 69.08 (17.70) | 67.87 (18.23) | <.001 |
| Respiratory rate, bpm | 18.89 (5.89) | 20.07 (5.94) | <.001 |
| Temperature, ℃ | 36.67 (0.79) | 36.80 (0.79) | <.001 |
| WBC, k/ul | 12.23 (9.29) | 13.48 (14.38) | <.001 |
| RBC, m/ul | 3.62 (0.78) | 3.52 (0.73) | <.001 |
| Hemoglobin, g/Dl | 10.83 (2.26) | 10.49 (2.12) | <.001 |
| Hematocrit, % | 32.78 (6.66) | 32.06 (6.32) | <.001 |
| RDW, % | 14.66 (2.22) | 15.19 (2.37) | <.001 |
| Platelet, k/ul | 203.58 (102.34) | 207.58 (109.79) | .016 |
| BUN, mg/dL | 24.00 (20.41) | 28.39 (23.54) | <.001 |
| Creatinine, mg/dL | 1.32 (1.48) | 1.43 (1.38) | <.001 |
| Calcium, mEq/L | 8.36 (0.80) | 8.25 (0.92) | <.001 |
| Potassium, mEq/L | 4.20 (0.70) | 4.10 (0.75) | <.001 |
| Sodium, mEq/L | 138.33 (5.03) | 138.20 (5.94) | .121 |
| Glucose, mg/dL | 142.88 (74.12) | 145.01 (75.14) | .076 |
| Chloride, mEq/L | 104.44 (6.34) | 104.02 (7.03) | <.001 |
| Aniongap, mEq/L | 13.96 (4.26) | 14.74 (4.17) | <.001 |
| INR | 1.43 (0.71) | 1.55 (0.92) | <.001 |
| PT, seconds | 15.69 (7.42) | 16.90 (9.88) | <.001 |
| PTT, seconds | 36.95 (21.65) | 38.28 (21.96) | <.001 |
| Myocardial infarct (%) | 8282 (17.1) | 652 (15.7) | .02 |
| Congestive heart failure (%) | 12007 (24.8) | 1172 (28.2) | <.001 |
| Cerebrovascular disease (%) | 8664 (17.9) | 829 (19.9) | .001 |
| Chronic pulmonary disease (%) | 11158 (23.1) | 982 (23.6) | .416 |
| Liver disease (%) | 5234 (10.8) | 591 (14.2) | <.001 |
| Renal disease (%) | 8740 (18.1) | 902 (21.7) | <.001 |
| Diabetes (%) | 13777 (28.5) | 1307 (31.4) | <.001 |
| Hypertension (%) | 21215 (43.8) | 1854 (44.6) | .349 |
| Malignant cancer (%) | 6184 (12.8) | 605 (14.6) | .001 |
| AIDS (%) | 242 (0.5) | 15 (0.4) | .263 |
| AKI (%) | 34583 (71.5) | 3011 (72.4) | .187 |
| AKI stage (%) |  |  | <.001 |
| 0 | 13814 (28.5) | 1146 (27.6) |  |
| 1 | 9453 (19.5) | 654 (15.7) |  |
| 2 | 17262 (35.7) | 1459 (35.1) |  |
| 3 | 7868 (16.3) | 898 (21.6) |  |
| Sepsis (%) | 22473 (46.4) | 2698 (64.9) | <.001 |
| Delirium (%) | 6888 (14.2) | 814 (19.6) | <.001 |
| Glucocorticoids systemic (%) | 6914 (14.3) | 527 (12.7) | .005 |
| ICS (%) | 2961 (6.1) | 266 (6.4) | .49 |
| Immunosuppressor (%) | 864 (1.8) | 80 (1.9) | .557 |
| Biologicals (%) | 37977 (78.5) | 3235 (77.8) | .339 |
| Vasopressors (%) | 16302 (33.7) | 1519 (36.5) | <.001 |
| PPIs (%) | 13970 (28.9) | 1416 (34.1) | <.001 |
| NMBA (%) | 1598 (3.3) | 144 (3.5) | .606 |
| Sedative (%) | 26811 (55.4) | 1937 (46.6) | <.001 |
| Opioids (%) | 17810 (36.8) | 1575 (37.9) | .168 |
| NSAIDs (%) | 29318 (60.6) | 2329 (56.0) | <.001 |
| Statins (%) | 14063 (29.1) | 925 (22.3) | <.001 |
| Invasive ventilation (%) | 19442 (40.2) | 1518 (36.5) | <.001 |
| Noninvasive ventilation (%) | 33375 (69.0) | 2819 (67.8) | .13 |
| CRRT (%) | 1732 (3.6) | 222 (5.3) | <.001 |
| Invasive lines (%) | 15137 (31.3) | 1089 (26.2) | <.001 |
| Tubes (%) | 1972 (4.1) | 183 (4.4) | .326 |
| Enteral Nutrition (%) | 6485 (13.4) | 859 (20.7) | <.001 |
| Parenteral Nutrition (%) | 490 (1.0) | 77 (1.9) | <.001 |
| antibiotic (%) | 29416 (60.8) | 2907 (69.9) | <.001 |
| Invasive ventilation hours | 26.38 (83.18) | 42.90 (128.80) | <.001 |
| Noninvasive ventilation hours | 25.45 (37.43) | 28.53 (44.77) | <.001 |
| CRRT days | 0.22 (1.74) | 0.40 (2.42) | <.001 |
| APSIII | 42.36 (20.10) | 48.70 (21.29) | <.001 |
| LODS | 4.24 (2.86) | 4.91 (2.99) | <.001 |
| OASIS | 31.07 (8.49) | 33.15 (8.38) | <.001 |
| SIRS | 2.55 (0.94) | 2.67 (0.93) | <.001 |
| SOFA | 4.32 (3.18) | 4.88 (3.44) | <.001 |
| SAPSII | 35.39 (13.91) | 39.57 (13.57) | <.001 |
| GCS | 14.87 (0.65) | 14.75 (0.84) | <.001 |
| Charlson comorbidity index | 4.78 (2.98) | 5.56 (2.92) | <.001 |
| Los hospital, days | 10.54 (11.14) | 16.23 (17.50) | <.001 |
| Hospital mortality (%) | 5055 (10.4) | 542 (13.0) | <.001 |
| Icu time 14d, days | 13.12 (2.89) | 13.20 (2.66) | .107 |
| Icu outcome 14d (%) | 4860 (10.0) | 443 (10.7) | .216 |
| Icu time 30d, days | 27.21 (7.66) | 26.99 (7.59) | .074 |
| Icu outcome 30d (%) | 6476 (13.4) | 655 (15.8) | <.001 |
| Icu time 90d, days | 77.62 (28.25) | 74.75 (29.80) | <.001 |
| Icu outcome 90d (%) | 8636 (17.8) | 973 (23.4) | <.001 |
| Icu time 365d, days | 293.06 (134.75) | 268.32 (146.04) | <.001 |
| Icu outcome 365d (%) | 11843 (24.5) | 1398 (33.6) | <.001 |

**Abbreviation:** SBP, systolic blood pressure; DBP, diastolic blood pressure; WBC, white blood cell; RBC, red blood cell; RDW, red cell distribution width; BUN, blood urea nitrogen; INR, international normalized ratio; PT, prothrombin time; PTT, partial thromboplastin time; AKI, acute kidney injury; ICS, inhaled corticosteroids; PPIs, proton pump inhibitors; NMBA, neuromuscular blocking agents; NSAIDs, non-steroidal anti-inflammatory drugs; CRRT, continuous renal replacement therapy; APSIII, Acute Physiology Score III; LODS, Logistic Organ Dysfunction Score; OASIS, Oxford Acute Severity of Illness Score; SIRS, systemic inflammatory response syndrome; SOFA, Sequential Organ Failure Assessment; SAPSII, Simplified Acute Physiology Score II; GCS, Glasgow Coma Scale; LOS, length of stay.


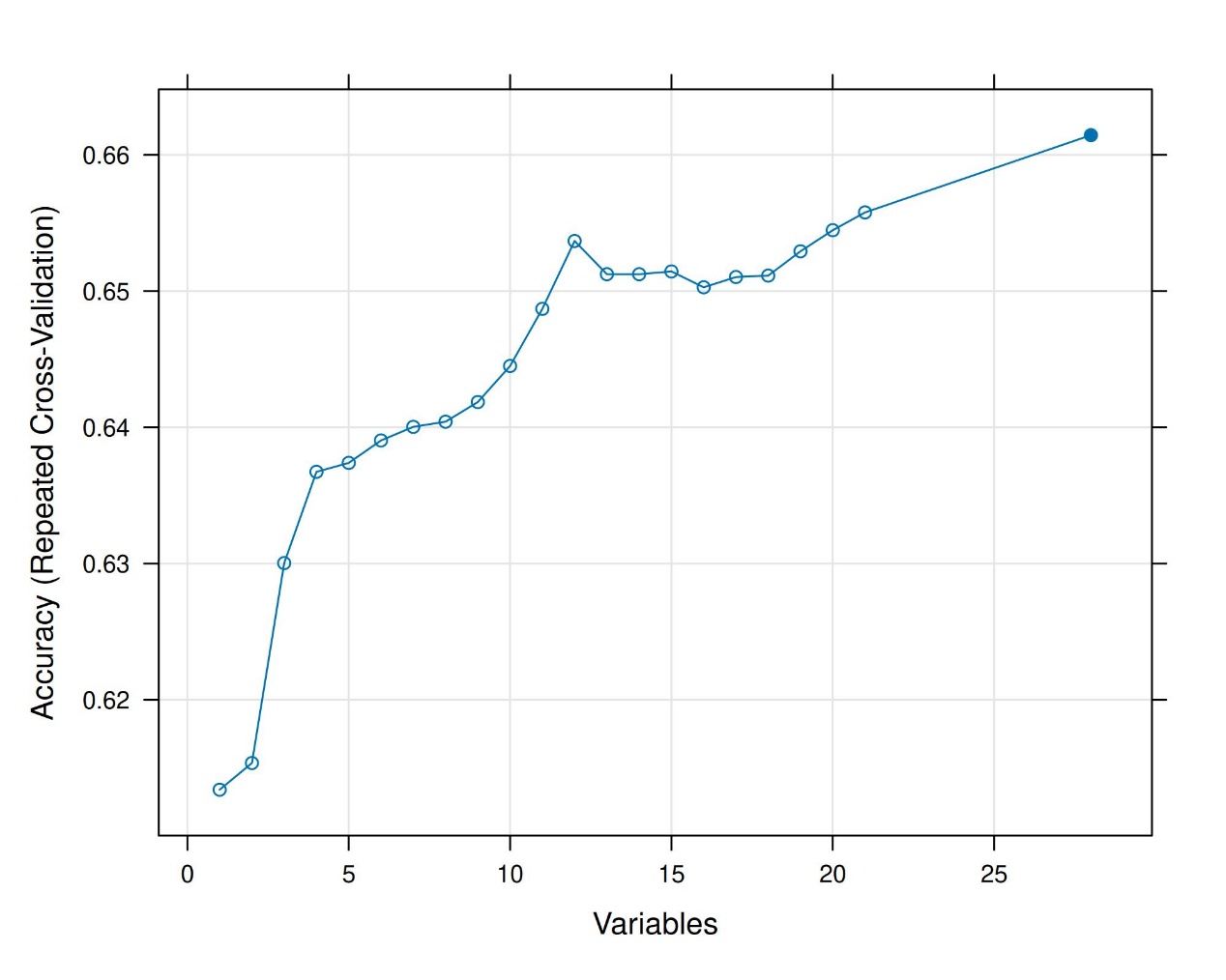


**Supplementary Figure 3** Performance of SVM model with different numbers of variables selected by recursive feature elimination.


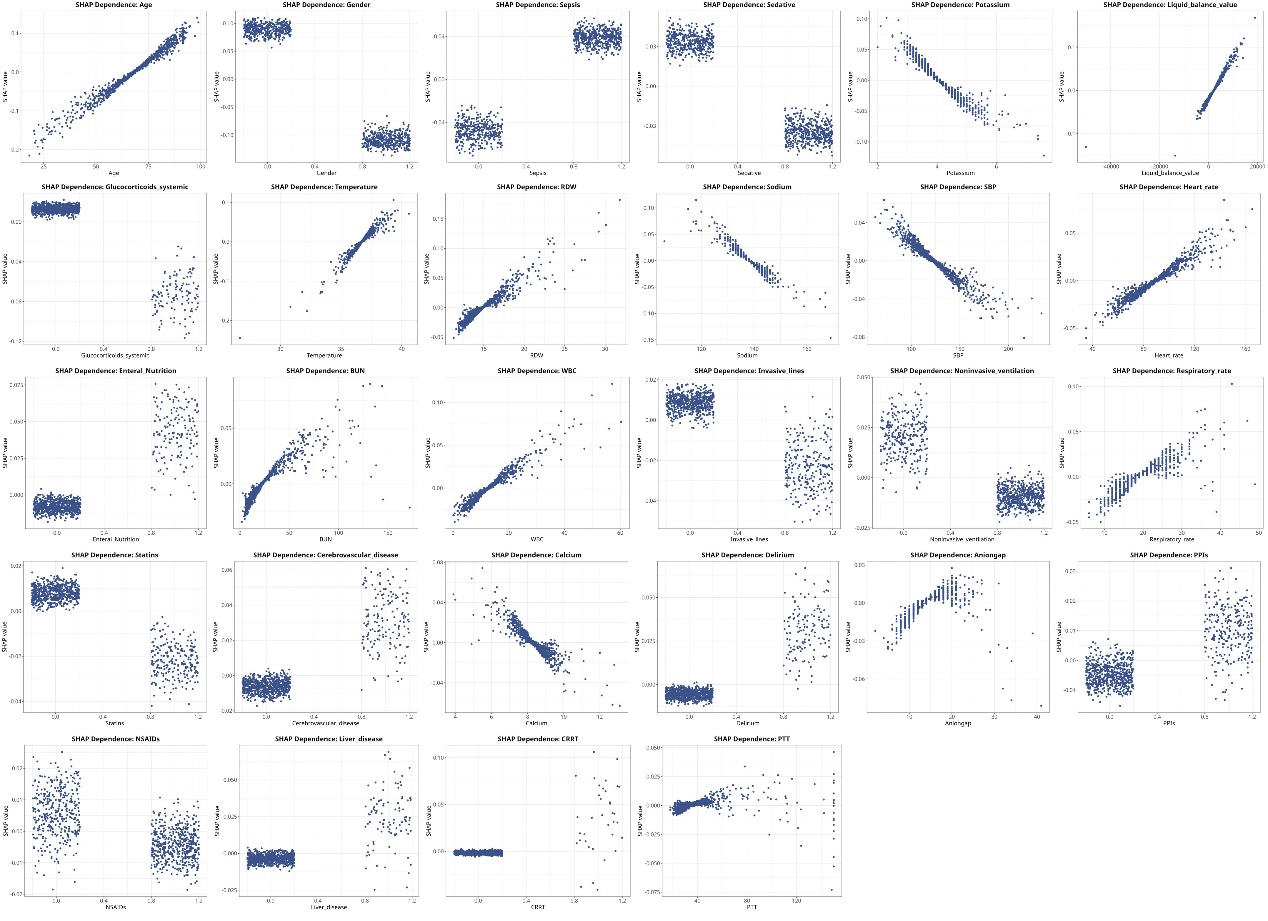


**Supplementary Figure 4** SHAP dependence plots for each selected feature in the SVM model. SHAP dependence plots for the 28 features identified by intersecting Boruta and LASSO selection, ranked by importance in the SVM model. The included variables are: Age, Gender, Sepsis, Sedative, Potassium, Liquid balance value, Glucocorticoids systemic, Temperature, RDW, Sodium, SBP, Heart rate, Enteral Nutrition, BUN, WBC, Invasive lines, Noninvasive ventilation, Respiratory rate, Statins, Cerebrovascular disease, Calcium, Delirium, Anion gap, PPIs, NSAIDs, Liver disease, CRRT, and PTT.


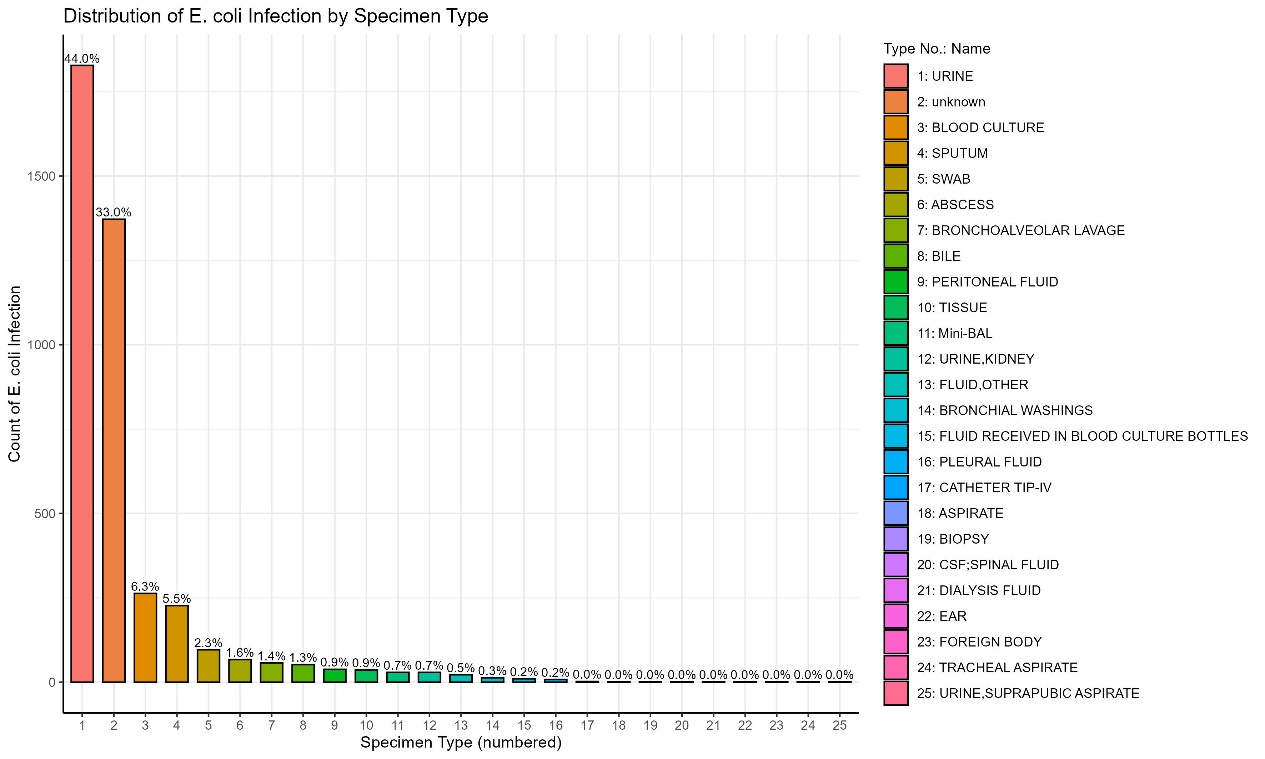


**Supplementary Figure 5** Distribution of *E. coli* infection by Specimen Type.
